# Rapid enrichment of mitochondria from mammalian cell cultures using digitonin

**DOI:** 10.1016/j.mex.2020.101197

**Published:** 2020-12-23

**Authors:** Bhavna Dixit, Shon Vanhoozer, Nana Abena Anti, Matthew S. O'Connor, Amutha Boominathan

**Affiliations:** Department of Mitochondrial Research, SENS Research Foundation, Mountain View, CA, 95051 USA

**Keywords:** Mitochondria purification, Chemical lysis of cells, Subcellular fractionation, Purity and integrity of mitochondria

## Abstract

We describe here a simple method to enrich mitochondrial fractions from mammalian cells for downstream analyses in the lab. Mitochondria purification involves cell lysis followed by separation of the organelles from the rest of the cellular components. Here, we use detergent to rupture the cell membrane of mammalian cells followed by differential centrifugation to enrich the organelles. Optimum conditions with respect to detergent concentration, time, sample size, and yield are discussed. The method's utility in downstream analyses and ease of processing multiple samples simultaneously is also described. All the reagents in this method can be assembled in-house, are economical, and are comparable, if not superior, to commercially available kits in terms of mitochondrial yield and integrity.

• Rapid enrichment of mitochondria from mammalian cells using commonly available reagents.

• Multiple samples can be processed simultaneously.

• Works over a wide range of sample size (1 million to 100 million cells).

## Graphical abstract

**Figure:**
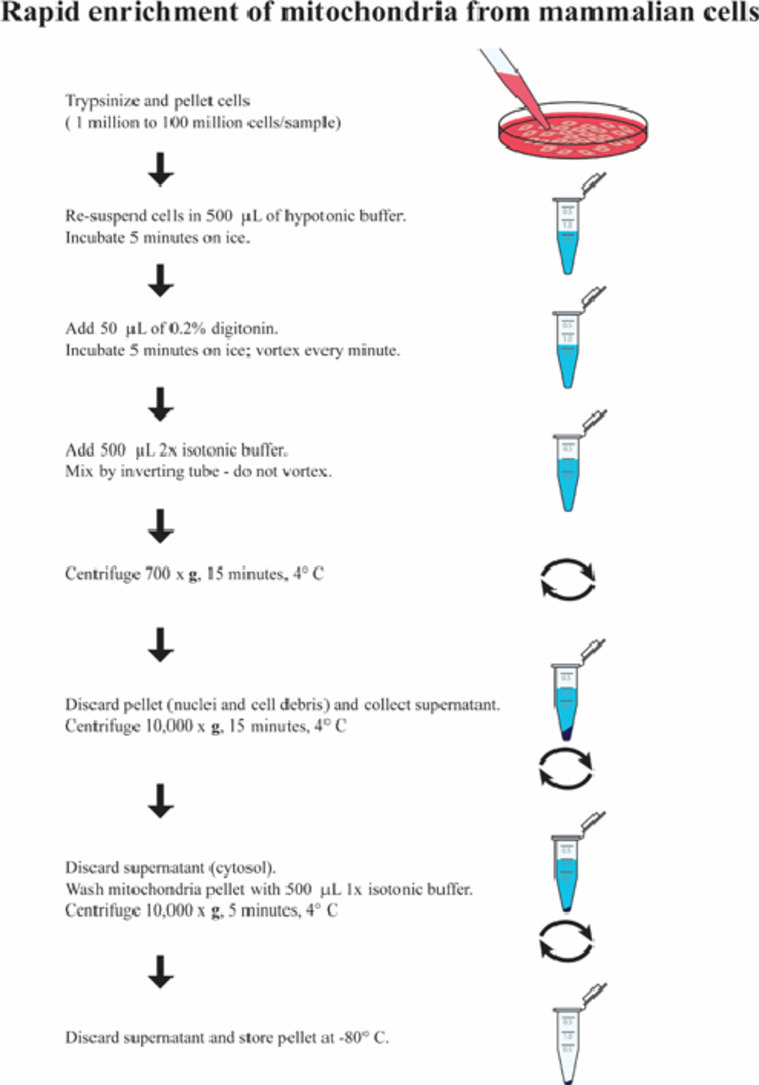

Specifications table

**Table:** 

Subject area:	*Biochemistry, Genetics and Molecular biology*
More specific subject area:	*Biochemistry*
Method name:	Purification of Mitochondria
Name and reference of original method:	Vercesi AE, Bernardes CF, Hoffmann ME, Gadelha FR, Docampo R. Digitonin permeabilization does not affect mitochondrial function and allows the determination of the mitochondrial membrane potential of Trypanosoma cruzi in situ. *J Biol Chem*. 1991;266(22):14431–14434.
Resource availability:	N/A

## Introduction

Mitochondria are cytoplasmic organelles (about 2 µm in size) that are involved in several critical processes in the cell, including the production of cellular ATP by oxidative phosphorylation. They are present in almost all eukaryotic cells and are enclosed by two membranes—an outer membrane and a tubular inner mitochondrial membrane that has a large surface area and encloses the matrix space [1]. They are also the only organelles in eukaryotes that contain genetic matter in addition to the nucleus, a remnant of the symbiotic relationship between an ancestral proteobacterium and a host eukaryotic cell. Functional analysis of these organelles in the laboratory often requires purification techniques to enrich/separate them from the rest of the cellular components. This is accomplished by disrupting the cell membrane followed by differential centrifugation. Cell disruption is typically achieved primarily by one of two methods: A) a time-consuming mechanical method, in which each sample is subjected to physical disruption (using Dounce homogenization [2]) or B) a relatively fast reagent-based method, in which cultured cells are lysed using commercially available proprietary formulations. In contrast, our method employs the mild non-ionic detergent digitonin [3,4] to permeabilize cell membranes of cultured cells in the process of purifying mitochondria. Digitonin permeabilizes lipid membranes by interacting with cholesterol. The plasma membrane has a high cholesterol content whereas mitochondria (particularly the inner mitochondrial membrane) has relatively low levels of cholesterol. This allows selective rupturing of the plasma membrane under controlled experimental conditions. Following permeabilization, the sample is processed via vortex mixing and just a few centrifugation steps. An early version of this method was utilized for samples in [5]. The final optimized version as described here was successfully used for data published in [6].

## Materials

### Cells

143B osteosarcoma cells were obtained from Dr. Carlos Moraes, University of Miami, Miami, FL, USA.

### Reagents

**Fig. 1:**
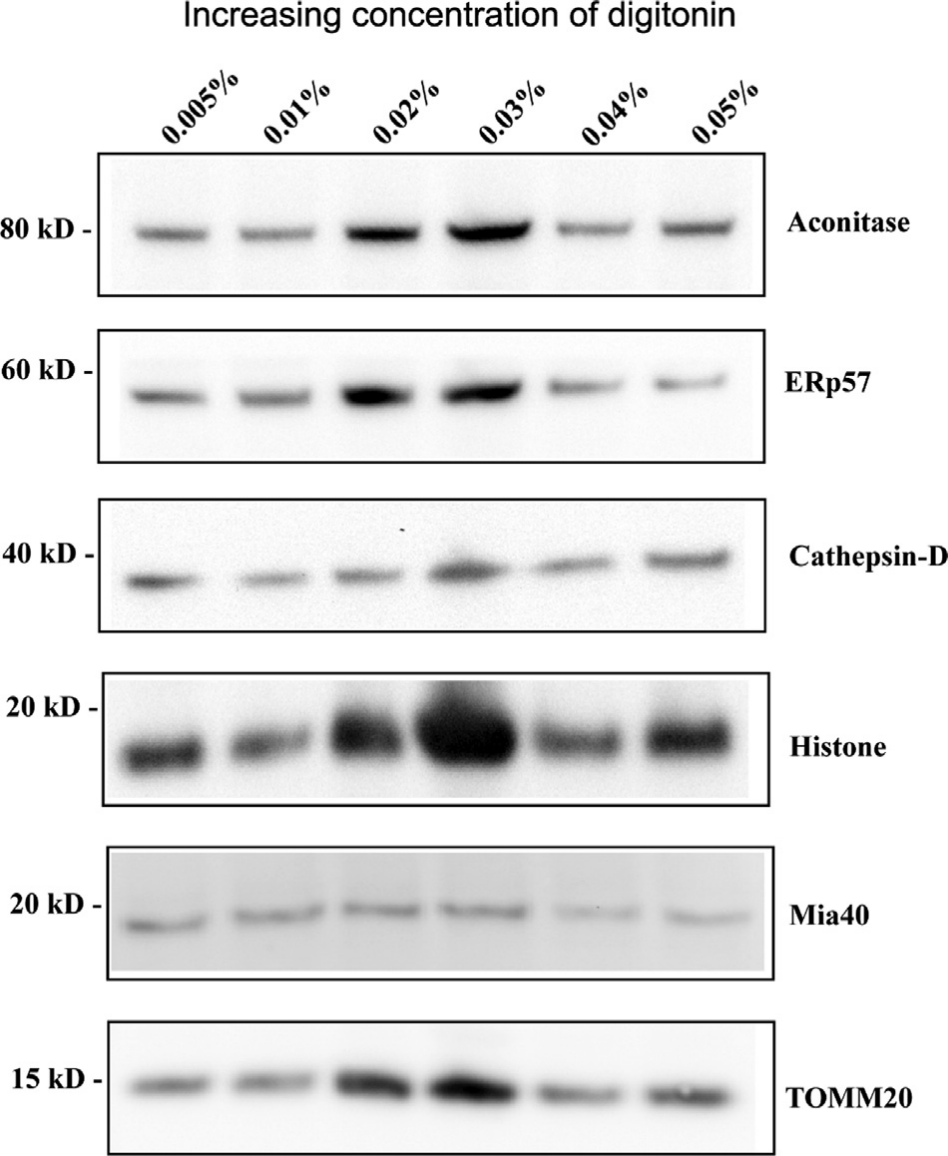
Optimum concentration of digitonin and mitochondrial yield. Equal volumes of purified mitochondrial fractions were resolved on 4–12% SDS PAGE gels and probed for Aconitase (mitochondrial matrix marker), Mia 40 (soluble intermembrane space marker), TOMM 20 (mitochondrial outer membrane marker) and other organelle markers: ERp57 (endoplasmic reticulum), Cathepsin D (lysosome) and Histone H3 (nuclear).

**Fig. 2:**
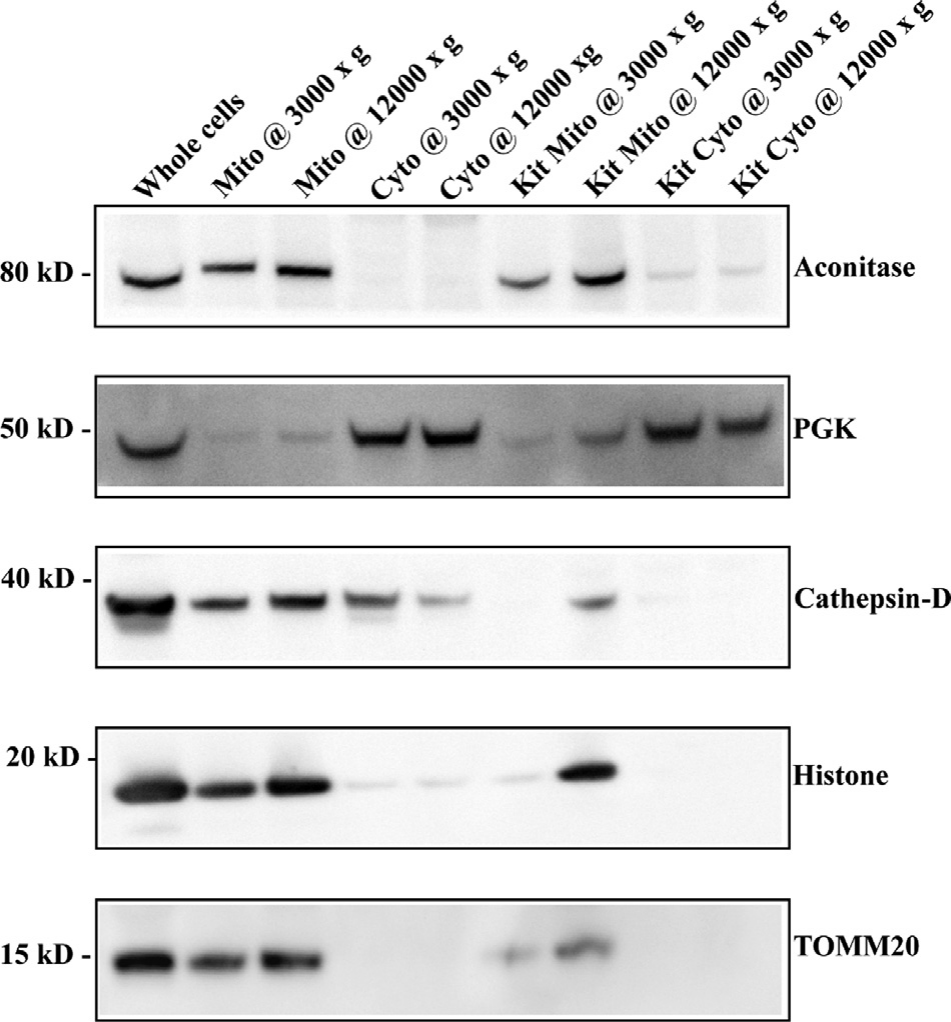
Comparison of mitochondrial yield and cytoplasmic contamination between the Optimized and Kit methods. Total cell lysates, mitochondria, and cytoplasmic fractions were normalized by volume and resolved on 4–12% SDS PAGE and probed for Aconitase (mitochondrial matrix marker), TOMM 20 (mitochondrial outer membrane marker) and other organelle markers: PGK (cytoplasm), Cathepsin D (lysosome) and Histone H3 (nuclear).

**Fig. 3:**
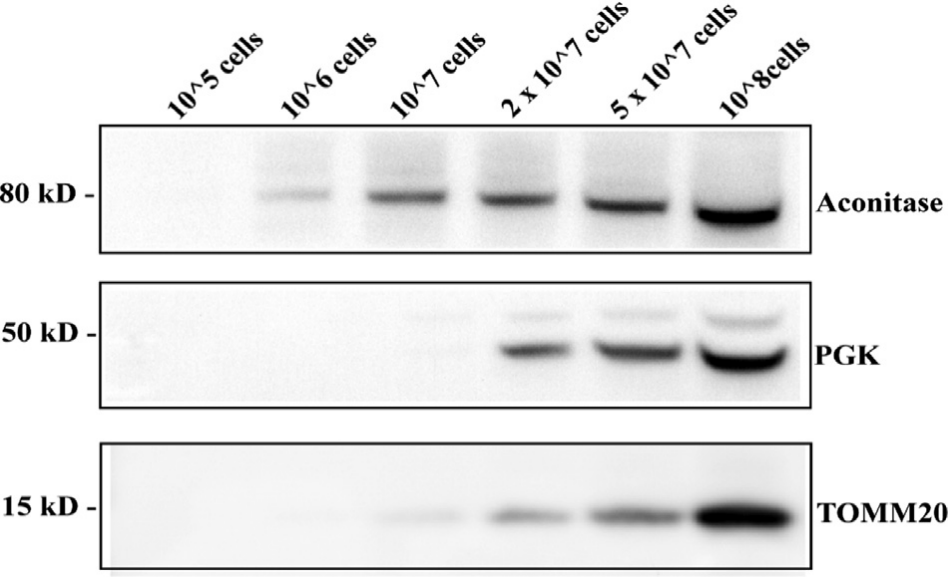
Mitochondrial yield as a function of sample size. Purified mitochondrial fractions were resolved on 4–12% SDS PAGE gels and probed for Aconitase (mitochondrial matrix marker), TOM 20 (mitochondrial outer membrane marker), and PGK (cytoplasm marker).

• Dulbecco's Modification of Eagle's Medium (DMEM Corning Cat#10-013-CV: 500 ml) with the following supplements: FBS, Tissue Culture Biologicals Cat#101: 50 ml; Uridine, Sigma Cat#U3003-5G: 50 µg/ml; Sodium pyruvate, ThermoFisher Cat#11360070: 5 ml; Antibiotic antimycotic solution 100X, VWR Cat#45000-616: 5 ml; Glutamax, Thermo scientific Cat#35050061: 5 ml
• Thermo Scientific™ Mitochondria Isolation Kit for Tissue. Cat#89801
• Corning® Dulbecco's Phosphate-Buffered Saline (DPBS), 1x without calcium and magnesium (21-031-CV)
• Corning® Trypsin EDTA 1x, 0.25 % Trypsin/2.21 mM EDTA in HBSS [-] sodium bicarbonate, calcium, magnesium, Porcine Parvovirus Tested (Corning 25-053-CI)
• Hypotonic buffer (155.02 mOsmol/L): 50 mM HEPES (1 M; pH = 7.0; Gibco; Cat#15630-080), 1 mM EDTA (0.5 M; pH 8.0; Millipore; Cat#324506), 1 mM Dithiothreitol (DTT, Cleland's reagent; VWR; Cat#97061-340), 1x Protease Inhibitor Cocktail (Sigma-Aldrich; Cat#P8340), 1 mM PMSF (Phenylmethanesulfonyl fluoride; Sigma-Aldrich; 78830), 1 mg/mL Bovine Serum Albumin (BSA), RPI Albumin, Bovine Fraction V (BSA);100 g Powder; Cat#A30075.100
• Isotonic buffer (2x): 100 mM HEPES (1 M; pH = 7.0; Gibco; Cat#15630-080), 2 mM Millipore EDTA (0.5 M; pH 8.0; Millipore; Cat#324506), 2 mM Dithiothreitol (DTT, Cleland's reagent; VWR; Cat#97061-340), 1x Protease Inhibitor Cocktail (Sigma-Aldrich; Cat#P8340 ), 2 mM PMSF (Phenylmethanesulfonyl fluoride; Sigma-Aldrich; 78830), 2 mg/mL Bovine Serum Albumin (BSA), RPI Albumin, Bovine Fraction V (BSA);100g Powder; Cat#A30075.100, 1.2 M D -Sorbitol (Sigma-Aldrich; Cat#50-70-4)
• Isotonic buffer (1x) (755.02 mOsmol/L): Dilute isotonic buffer (2x) 1:1 with RO water
• Digitonin (Sigma-Aldrich; Cat#D141)
• Antibodies and their dilutions:

1. Anti-Aconitase 2 (ACO2) mouse monoclonal antibody [Clone ID: OTI3G8]; Origene; Cat#TA500824; dilution 1:1000 (Fig. 1) and 1:500 (Fig. 2, Fig. 3)
2. Anti-TOMM20 (TOMM20) rabbit polyclonal antibody; Abclonal; Cat#A6774; dilution 1:1000 (Fig. 1) and 1:500 (Fig. 2 and 3)
3. Anti-Phosphoglycerate kinase (PGK1) rabbit polyclonal antibody; Abcam; Cat#14039; dilution 1:500 (Fig. 2 and 3)
4. Anti-Cathepsin D mouse monoclonal antibody; Novus Biologicals; Cat#NBP1-04278; dilution 1:1000 (Fig. 1) and 1:500 (Fig. 2 and 3)
5. Anti-Histone H3 rabbit antibody; Sigma-Aldrich; Cat#H0164-25UL; dilution 1:1000 (Fig. 1) and 1:500 (Fig. 2 and 3)
6. Anti-ERp57/PDIA3 mouse antibody; Novus Biologicals; Cat#NBP2-59695-25µg; dilution 1:1000 (Fig. 1) and 1:500 (Fig. 2 and 3)
7. Anti-Mia40/CHCHD4 rabbit antibody; LSBio; Cat#LS-C802857-100; dilution 1:200 in 3% milk (Fig. 1)

### Equipment

Centrifuge tubes, a swinging bucket tabletop centrifuge (Eppendorf, Hamburg, Germany), microfuge, vortex mixer, Invitrogen Countess™ Automated Cell Counter Cat# C10227.

### Procedure

1. All the steps were performed on ice.
2. Count and harvest cells (1 × 10^6^ to 1 × 10^8^ cells per sample) by trypsinization. Cells were counted using the Invitrogen™ Countess™ Automated Cell Counter.
3. Wash cells with 1X PBS and pellet at 215 × **g**, 5 min at 4 °C.
4. Resuspend pelleted cells in 500 µl of hypotonic buffer and incubate on ice for 5 min.
5. For cell lysis, add 50 µl of 0.2 % high purity digitonin to achieve a final concentration of 0.02%. Vortex vigorously (5–10 s) every minute for 5 min.
6. Add 1:1 volume (500 µl) of 2x isotonic buffer to the samples and centrifuge immediately in a benchtop microfuge at 700 × **g** for 10 min at 4 °C to remove unbroken cells and cell debris.
7. Carefully transfer the supernatant to a new tube and centrifuge at 10,000 × **g** for 15 min at 4 °C. Remove the supernatant (cytoplasmic fraction).
8. For washing the mitochondria-containing pellet, add 500 µl of 1x isotonic buffer on top of the pellet. Do not resuspend; instead, invert the position of the pellet in the centrifuge and spin at 10,000 × **g** for 5 min at 4 °C.
9. Aspirate the supernatant completely and store the mitochondria-containing pellet at -80 °C until further use.

### Notes

1. Hypotonic and isotonic buffer stock solutions are prepared without Protease Inhibitor Cocktail and PMSF. Add Protease Inhibitor Cocktail and PMSF to buffers before starting each experiment.
2. Digitonin must be prepared fresh for each experiment.
3. The number of mitochondria per cell varies from cell type to cell type, hence the number of cells required for optimum yields can also vary and must be standardized.
4. Commercially available kits require 2 × 10^7^ cultured cells as starting material for isolation of mitochondria whereas our in-house optimized method can process varying numbers of cells ranging from 1 × 10^6^ to 1 × 10^8^ cells.
5. For immunodetection of Mia40, samples were incubated with 5-5′-Dithiobis(2-nitrobenzoic acid (DTNB) at 1 mM for 30 min at room temperature before resolving on SDS PAGE gels.

## Results

This protocol was standardized for 143B osteosarcoma cells, however, it can be adapted to other mammalian cell lines. Discussed below are optimum conditions with respect to the number of cells, digitonin concentration, yield and purity of mitochondria, and levels of co-contamination from other organelles. Mitochondria enriched samples were routinely analyzed by SDS PAGE, transferred onto PVDF membranes and immunodetected for various cellular markers.

### Optimum digitonin concentration for cell lysis

143B cells (2 × 10^7^ cells per sample) were incubated with increasing concentrations of digitonin (0.005%, 0.01%, 0.02%, 0.03%, 0.04% and 0.05%), and processed as described in the methods section (*n* = 3). Mitochondrial fractions (one-third of the total volume) were resolved on 4-12% SDS PAGE gels and immunodetected for Aconitase (mitochondrial matrix marker), Mia40 (mitochondrial intermembrane space marker), TOMM 20 (mitochondrial outer membrane marker) and other organelle markers: ERp57 (endoplasmic reticulum), Cathepsin D (lysosome) and Histone H3 (nuclear). 0.02% digitonin was the optimum concentration in terms of mitochondrial yield and minimal cytoplasmic contamination as seen in Fig. 1. While there were appreciable yields at 0.02 and 0.03% digitonin, the co-purifying nuclear fraction was higher at 0.03% digitonin. Furthermore, the immunodetection profiles for an outer membrane marker (TOMM20) and a matrix marker (Aconitase) are identical at 0.02 % and 0.03% digitonin. This indicates that the inner membrane and its contents (matrix) are preserved in the purification procedure up to 0.03% digitonin, however, this does not address the integrity of the outer membrane. In order to evaluate the outer membrane integrity, we assessed the levels of a soluble intermembrane space protein, Mia40 [7]. As observed in Fig. 1 (panel 5 from top) Mia40 levels are also maintained up to 0.03% digitonin. Concentrations ≥ 0.04% digitonin tend to lyse the subcellular components, and thus Mia40 levels were reduced as expected.

### Comparison of yield and co-purifying fractions between the optimized method and commercial kit protocols

Mitochondria and cytoplasmic fractions were isolated from 143B cells (2 × 10^7^ cells per sample) using the optimized protocol as described in the methods sections, or by following the manufacturer's instructions for the commercial kit (ThermoFisher Scientific Mitochondria Isolation Kit for Cultured Cells, Cat#89874). Both these methods yield crude mitochondrial preparations with varying degrees of co-purifying organelles such as lysosomes and endoplasmic reticulum. Therefore, a preclearing spin was introduced at 3,000 × **g** before the final high-speed spin to reduce co-purifying contaminants. Total cells, mitochondria and cytoplasmic fractions for both the post nuclear centrifugation spins 3,000 × **g** and 12,000 × **g** were run on 4–12% SDS PAGE (one-third of the total volume in every case) and probed for various markers: Aconitase (mitochondrial matrix), TOMM 20 (mitochondrial outer membrane), PGK (cytosol), ERp57 (endoplasmic reticulum), Cathepsin D (lysosome) and Histone H3 (nuclear marker). Higher mitochondrial yield and less cytoplasmic contamination was observed in samples isolated using the optimized method (Fig. 2). The commercial kit protocol recommends a 12,000 × **g** final spin to maximize yield vs a 3,000 × **g** final spin to maximize purity. With our method, higher centrifugation speeds also yielded more mitochondria and the copurifying organelles were also enriched.

### Sample size

We assessed the efficiency of our method at 0.02% digitonin concentration for cell lysis as a function of sample size i.e., the number of cells. Equivalent volumes of fractions containing enriched mitochondria were analyzed on 4-12% SDS PAGE gels and probed for various markers. As evident from Fig. 3, a wide range of starting cell numbers can be employed to effectively isolate mitochondria (1 × 10^6^ to 1 × 10^8^). However, at cell numbers > 2 × 10^7^, the cytoplasmic contamination increased significantly.

## Conclusions

We developed a quick and cost-effective method for the isolation of mitochondria from mammalian cells. The method uses common and inexpensive reagents and could be adapted to other cell lines for specific research conditions. Despite multiple centrifugation steps, it takes ~40 minutes to isolate mitochondria from harvested cells. Under the protocol time frames, it is possible to process up to 10 samples simultaneously. We believe that this method could be adapted for use in tissue samples by titrating for optimum digitonin concentration, but we have not yet attempted this application.

## Sources of funding

We thank SENS Research Foundation, Forever Healthy Foundation, Foster Foundation and LifeSpan.io for financial support.

## Author contribution statement

BD, MSO and AB designed the experiments, BD, SV and NAA performed the experiments, BD, MSO and AB analyzed the data and wrote the manuscript. All authors were involved in editing.
